# Studying Synaptic Vesicle Pools using Photoconversion of Styryl Dyes

**DOI:** 10.3791/1790

**Published:** 2010-02-15

**Authors:** Felipe Opazo, Silvio O. Rizzoli

**Affiliations:** STED Microscopy of Synaptic Function, European Neuroscience Institute Göttingen

## Abstract

The fusion of synaptic vesicles with the plasma membrane (exocytosis) is a required step in neurotransmitter release and neuronal communication. The vesicles are then retrieved from the plasma membrane (endocytosis) and grouped together with the general pool of vesicles within the nerve terminal, until they undergo a new exo- and endocytosis cycle (vesicle recycling). These processes have been studied using a variety of techniques such as electron microscopy, electrophysiology recordings, amperometry and capacitance measurements. Importantly, during the last two decades a number of fluorescently labeled markers emerged, allowing optical techniques to track vesicles in their recycling dynamics. One of the most commonly used markers is the styryl or FM dye ^1^; structurally, all FM dyes contain a hydrophilic head and a lipophilic tail connected through an aromatic ring and one or more double bonds (Fig. 1B). A classical FM dye experiment to label a pool of vesicles consists in bathing the preparation (Fig. 1Ai) with the dye during the stimulation of the nerve (electrically or with high K^+^). This induces vesicle recycling and the subsequent loading of the dye into recently endocytosed vesicles (Fig. 1A_i-iii_). After loading the vesicles with dye, a second round of stimulation in a dye-free bath would trigger the FM release through exocytosis (Fig. 1A_iv-v_), process that can be followed by monitoring the fluorescence intensity decrease (destaining).

Although FM dyes have contributed greatly to the field of vesicle recycling, it is not possible to determine the exact localization or morphology of individual vesicles by using conventional fluorescence microscopy. For that reason, we explain here how FM dyes can also be used as endocytic markers using electron microscopy, through photoconversion. The photoconversion technique exploits the property of fluorescent dyes to generate reactive oxygen species under intense illumination. Fluorescently labeled preparations are submerged in a solution containing diaminobenzidine (DAB) and illuminated. Reactive species generated by the dye molecules oxidize the DAB, which forms a stable, insoluble precipitate that has a dark appearance and can be easily distinguished in electron microscopy ^2,3^. As DAB is only oxidized in the immediate vicinity of fluorescent molecules (as the reactive oxygen species are short-lived), the technique ensures that only fluorescently labeled structures are going to contain the electron-dense precipitate. The technique thus allows the study of the exact location and morphology of actively recycling organelles.

**Figure:**
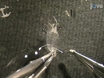


## Protocol

### 1) Preparation of *Drosophila melanogaster* neuronal muscular junction (NMJ)

Prepare standard Drosophila saline (130 mM NaCl, 36 mM sucrose, 5 mM KCl, 2 mM CaCl_2_, 2 mM MgCl_2_, 5 mM Hepes, pH 7.3 ^4^.
Dissect the preparation in saline (1.1). The Drosophila larvae is pined dorsal side-up in a Sylgard dish; the dorsal side is sectioned longitudinal, and the internal organs are removed. The preparation is then stretched and pined. Several ventral muscles can be then used.

### 2) Stimulation and FM staining

It is advisable to do all the following steps in low light conditions, in order to protect FM dyes from bleaching.
For chemical stimulation of the nerves, high potassium buffer is used. Prepare Drosophila saline (see 1.1) containing 90 mM of KCl and 10 μM of FM1-43 dye. Keep the solution protected from light.
Bath the preparation with the previously prepared buffer for 1 minute at room temperature.
Wash with standard Drosophila saline (1.1) to remove extracellular FM1-43 dye. Proceed rapidly to fixation.

### 3) Fixing

Bath the preparation with 2.5% glutaraldehyde in phosphate-buffered saline (PBS) for 45 minutes at room temperature. For good preservation of morphological features, glutaraldehyde is preferred to paraformaldehyde.
Wash once with PBS and then leave the preparation submerged for 15 minutes in 100 mM of NH_4_Cl. This step is done in order to quench the free aldehyde groups of the remaining glutaraldehyde fixative.
Wash the NH_4_Cl solution out with normal PBS.

### 4) Photoconversion

Incubate the NMJ preparation for 30 minutes at 4°C in PBS containing 1.5 mg ml-1 of diaminobenzidine (DAB).
Position the sample under the fluorescent microscope. Find and focus the fluorescent signal using a relatively low magnification water immersion objective (20x 0.5 NA).
Illuminate the sample with the maximum lamp intensity until the FM dye is completely bleached (Figure 1C). To control if photoconversion has occurred, is advisable to check at the sample under transmission light before and after the FM dye-bleaching step. If photoconversion takes place, a dark brown precipitate is expected (Figure 1C). The illumination time is variable, depending upon the illumination strength and also upon the DAB penetration into the preparation. For most preparations, we found illumination times of ~30-45 minutes to be optimal when using a 20x objective. Shorter illumination periods are used for higher magnification objectives (60x).
For illumination we prefer to use a mercury lamp, with a lamp housing containing a back mirror, which collects the back-scattered light beams, and therefore increases total intensity.

### 5) Processing the sample for EM

Osmication
  Prepare a solution with one volume of osmium tetroxide in tree volumes of water (approx. 300 μl per sample). Work using osmium tetroxide must be done under the hood, using gloves and aye protection equipment.
  Incubate the preparation in the osmium tetroxide solution for 1 hour at room temperature (Under the hood).
  Wash extensively with PBS (4-5 times 5 minutes) and transfer each samples to a clean glass flask.
Dehydration
  Prepare separate solutions containing 30, 50, 70 90, 95 and 100% of ethanol.
  Add 1 ml of 30% ethanol to each sample for 5 minutes.
  Add 1 ml of 50% ethanol to each sample for 5 minutes.
  Add 1 ml of 70% ethanol to each sample for 5 minutes.
  Add 1 ml of 90% ethanol to each sample for 5 minutes.
  Add 1 ml of 95% ethanol to each sample for 5 minutes (repeat 3x).
  Add 1 ml of 100% ethanol to each sample for 5 minutes (repeat 3x).
Embedding and further processing
  Mix 1 volume of Epon resin with one volume of ethanol. Add it to the preparation under continuous rotation (2-4 hours).
  Place the preparation in 100% Epon in open flasks, allow the remaining ethanol to evaporate (4-6 hours).
  Place the preparation in moulds at 60°C for 36 hours.
  Electron microscopy processing. Process the preparation in 50-80 nm thin sections, using standard ultramicrotomy procedures.
Electron microscopy imaging
  The preparation is imaged using conventional EM procedures.

### 6) Representative Results

The expected results are outlined in Figures 1C and 2. The illumination procedure results in the formation of brown DAB precipitate, which will be visible in both fluorescence and transmission imaging. The first occurrence during illumination is the disappearance of fluorescence associated with the FM dye staining. The background fluorescence induced by the aldehyde fixative, in contrast, will be visible throughout the experiment. The bleaching takes place typically in ~10-20 minutes after the start of illumination. Typically no photoconversion product can be observed at this time point.

Illumination should be continued, and after ~10 minutes the preparations will turn to a brown shade due to DAB accumulation (Fig 1C_iv_) It is unclear why DAB precipitation and FM dye bleaching do not take place simultaneously; it is possible that a relatively large amount of oxidized DAB needs to accumulate before aggregation and precipitation, and that therefore this reaction is slower than the bleaching process. At this stage, however, the preparation is not ready for electron microscopy processing, as the DAB precipitation is likely incomplete. We prefer to wait 5-10 minutes longer, during which the preparation (presynaptic nerve terminal) assumes a dark-brown (or black) color, indicative of complete conversion. A slight conversion of the general surface of the preparation (for example, of the muscle fibers in a neuromuscular junction) can be observed at this stage. It is most likely related to a conversion of DAB induced by autofluorescence (and/or fixative fluorescence), and it is not detrimental to the procedure.

The preparation is then processed for electron microscopy, and should be checked for the presence of dark (labeled) vesicles. As we have demonstrated before ^5,6^, these vesicles are much denser than non-labeled ones, and are therefore easily distinguished (Figure 2B). To increase the chance of distinguishing well the different types of vesicles, no contrast-enhancing post staining of the sections should be performed (no uranyl acetate or lead citrate staining); the osmium staining is sufficient to observe most cellular elements, and is not as dark as DAB precipitate.


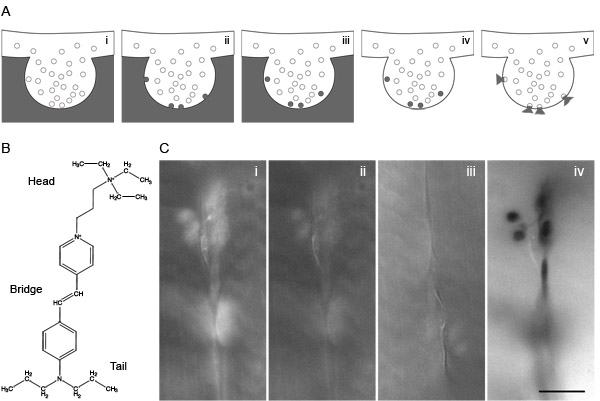
 **Figure 1: FM dyes and it use for photoconversion**. (**A**) Represent the steps of a classical experiment for loading and distaining vesicles using FM dye under stimulation. (**i**) Incubate the sample in FM dye containing buffer. (**ii**) Stimulate (electrically or chemically) to induce vesicle fusion in presence of FM dye. (**iii**) The subsequence endocytosis after stimulation will load some vesicles with FM dye still present in the extracellular buffer. (**iv**) Replace the extracellular FM dye by washing with buffer. (**v**) A new stimulation will induce loaded vesicles to release their FM dye upon exocytosis. (**B**) Schematic showing the head, bridge and tail of the FM1-43 dye. (**C**) Example of a photoconversion experiment in *Drosophila* NMJ. (**i**) After loading the NMJ with FM1-43, washing external dye molecules and fixing, the fluorescence of a nerve terminal can be observed with a conventional epifluorescence microscope (63x). (**ii-iii**) Under continuous illumination the dye is completely bleached. (**iv**) When illumination continues, a black color appears due to diaminobenzidine precipitation. Scale bar represents 10 μm.


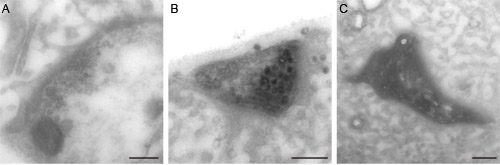
 **Figure 2: EM examples of photoconverted samples. **(**A**) The image shows a nerve terminal containing synaptic vesicles, but not of them are photoconverted; this might be caused by not having enough illumination or poor diaminobenzidine penetration in the tissue. (**B**) Synaptic bouton with several dark (filled) vesicles that went through FM photoconversion. (**C**) Excess of illumination results in an overall dark terminal where no vesicles or organelles are distinguishable. Scale bars represent 200 nm.

## Discussion

A few critical steps should be taken into account:

The DAB incubation should be performed only after thorough washing and quenching of the preparations. Otherwise the non-reacted glutaraldehyde will interact with DAB and cause its precipitation (typically in the form of flat crystals, which are not electron dense). Preparations where this precipitation takes place abundantly are rarely usable for electron microscopy.
Illumination times should be optimized, by testing several identical preparations with different illumination times. In our experience, optimal conversion (Figure 2B) is achieved in a time window of about 5 minutes (for example, between 35 and 40 minutes after the start of illumination). Insufficient illumination (Figure 2A) is characterized by a lack of labeled organelles, and/or partially labeled organelles (such as vesicles appearing to contain DAB in only half of their volume). Long illumination, in contrast, results in over-conversion   the DAB conversion product accumulates, distorts the organelles and escapes into the cytosol (Figure 2C). The preparations appear as black structures in electron microscopy, with little observable morphology.

We have used the technique in several preparations, including neuromuscular junctions from *Caenorhabditis elegans*, *Drosophila melanogaster*, zebrafish (*Danio rerio*) and frog (*Rana pipiens* and *Rana esculenta*). We have also used the technique in cultured cells, including cultured neurons and neuroendocrine cells. It can also be used successfully in purified synapses from rat brain (synaptosomes). Therefore, we expect the technique to be usable in most preparations which use vesicular membrane uptake. The technique it is the most sensitive technique to date in determining the positioning and morphology of endocytosed organelles. It was used to determine the exact location of recently endocytosed organelles, including specific physiological pools of vesicles ^5,6,7,8^. It has also been used in determining the number and morphology of the organelles in the recycling pathway see for example ^9,10^. 

## References

[B0] Betz WJ, Bewick GS (1992). Optical analysis of synaptic vesicle recycling at the frog neuromuscular junction. Science.

[B1] Henkel AW, Lübke J, Betz WJ (1996). FM1-43 dye ultrastructural localization in and release from frog motor nerve terminals. Proc Natl Acad Sci USA.

[B2] Sandell JH, Masland RH (1988). Photoconversion of some fluorescent markers to a diaminobenzidine product. J Histochem Cytochem.

[B3] Kuromi H, Kidokoro Y (1999). The optically determined size of exo/endo cycling vesicle pool correlates with the quantal content at the neuromuscular junction of Drosophila larvae. J Neurosci.

[B4] Denker A, Kröhnert K, Rizzoli SO (2009). Revisiting synaptic vesicle pool localization in the Drosophila neuromuscular junction. J Physiol (Lond).

[B5] Rizzoli SO, Betz WJ (2004). The structural organization of the readily releasable pool of synaptic vesicles. Science.

[B6] Darcy KJ, Staras K, Collinson LM, Goda Y (2006). Constitutive sharing of recycling synaptic vesicles between presynaptic boutons. Nat Neurosci.

[B7] Harata N, Ryan TA, Smith SJ, Buchanan J, Tsien RW (2001). Visualizing recycling synaptic vesicles in hippocampal neurons by FM1-43 photoconversion. Proc Natl Acad Sci USA.

[B8] Lange RPJde, de Roos ADG, Borst JGG (2003). Two modes of vesicle recycling in the rat calyx of Held. J Neurosci.

[B9] Richards DA, Guatimosim C, Rizzoli SO, Betz WJ (2003). Synaptic vesicle pools at the frog neuromuscular junction. Neuron.

